# Overlapping effector interfaces define the multiple functions of the HIV-1 Nef polyproline helix

**DOI:** 10.1186/1742-4690-9-47

**Published:** 2012-05-31

**Authors:** Lillian S Kuo, Laura L Baugh, Sarah J Denial, Richard L Watkins, Mingjie Liu, J Victor Garcia, John L Foster

**Affiliations:** 1 Division of Infectious Diseases, Center for AIDS Research, University of North Carolina, Chapel Hill, NC, 27599-7042, USA; 2 Department of Internal Medicine, University of Texas Southwestern Medical Center at Dallas, 5323 Harry Hines Boulevard, Y9.206, Dallas, TX, 75390, USA

**Keywords:** HIV-1, Nef, CD4, MHC class I, p21-activated protein kinase, Protein-protein interaction interface, SH3 domain

## Abstract

**Background:**

HIV-1 Nef is a multifunctional protein required for full pathogenicity of the virus. As Nef has no known enzymatic activity, it necessarily functions through protein-protein interaction interfaces. A critical Nef protein interaction interface is centered on its polyproline segment (P_69_VRPQVPLRP_78_) which contains the helical SH3 domain binding protein motif, PXXPXR. We hypothesized that any Nef-SH3 domain interactions would be lost upon mutation of the prolines or arginine of PXXPXR. Further, mutation of the non-motif “X” residues, (Q73, V74, and L75) would give altered patterns of inhibition for different Nef/SH3 domain protein interactions.

**Results:**

We found that mutations of either of the prolines or the arginine of PXXPXR are defective for Nef-Hck binding, Nef/activated PAK2 complex formation and enhancement of virion infectivity (EVI). Mutation of the non-motif “X” residues (Q, V and L) gave similar patterns of inhibition for Nef/activated PAK2 complex formation and EVI which were distinct from the pattern for Hck binding. These results implicate an SH3 domain containing protein other than Hck for Nef/activated PAK2 complex formation and EVI. We have also mutated Nef residues at the N-and C-terminal ends of the polyproline segment to explore interactions outside of PXXPXR. We discovered a new locus GFP/F (G_67_, F_68_, P_69_ and F_90_) that is required for Nef/activated PAK2 complex formation and EVI.

MHC Class I (MHCI) downregulation was only partially inhibited by mutating the PXXPXR motif residues, but was fully inhibited by mutating the C-terminal P_78_. Further, we observed that MHCI downregulation strictly requires G_67_ and F_68_. Our mutational analysis confirms the recently reported structure of the complex between Nef, AP-1 μ1 and the cytoplasmic tail of MHCI, but does not support involvement of an SH3 domain protein in MHCI downregulation.

**Conclusion:**

Nef has evolved to be dependent on interactions with multiple SH3 domain proteins. To the N- and C- terminal sides of the polyproline helix are multifunctional protein interaction sites. The polyproline segment is also adapted to downregulate MHCI with a non-canonical binding surface. Our results demonstrate that Nef polyproline helix is highly adapted to directly interact with multiple host cell proteins.

## Background

HIV-1 Nef is a highly significant pathogenic factor, but its role in the progression to AIDS is not mechanistically understood. Nef is remarkable in the number and diversity of its functions, and its pathogenicity may depend on the collective impact of its highly conserved multiple activities [1,2]. Among its numerous activities, Nef downregulates major histocompatibility complex class I (MHCI) from the cell surface, downregulates CD4, activates and forms a complex with p21-activated protein kinase 2 (PAK2) and enhances the infectivity of the HIV-1 virion [2,3]. It is plausible that each of these four Nef functions makes contributions to HIV-1 Nef pathogenicity. Nef/activated PAK2 complex formation may enhance cellular activation and, as a result, viral replication [4]. MHCI downregulation appears to blunt the host cytotoxic T cell response to the virus [5-7]. CD4 downregulation may enhance the efficiency of viral production [8-10]. Finally, enhancement of virion infectivity (EVI) may facilitate the spread of the virus throughout the infected patient [11].

Nef downregulates or upregulates a large number of cell surface receptors, though it has recently been suggested that the mechanism of modulating the levels of many of these proteins is indirect [12,13]. The mechanisms of these indirect effects are, in general, unknown; and ultimately Nef must directly interact with host cell proteins as it has no enzymatic activity [2]. Here, we have studied Nef activities that appear to depend on direct interactions between Nef and relevant host cell target proteins. These activities include CD4 and MHCI downregulation, as direct binding of Nef to the cytoplasmic tails of these membrane proteins has been demonstrated [14-19]. Also, direct roles for SH3 domain proteins have been suggested for several Nef activities: 1) the SH3 domain protein, Hck, is known to bind Nef with nM affinity [20]; 2) Activation of Hck and other Src family kinases has been proposed to facilitate MHCI downregulation by formation of a Nef/Src family kinase/ ZAP70/PI3K complex [21,22]; 3) The activation of PAK2 and the formation of the Nef/activated PAK2 complex appear to require an SH3 domain protein [20]. Finally, mutational evidence suggests the involvement of an SH3 domain containing protein for EVI [2].

To begin to unravel the complex interactions between Nef and the multiple host cell proteins involved in these Nef activities, we have used fine structure mutational analysis to describe the roles of the polyproline segment, P_69_VRPQVPLRP_78_, residues. First, we mutated each residue of the core sequence, PQVPLR, that represents the Nef SH3 domain binding motif (PXXPXR) [23,24]. Our analysis confirmed the importance of the prolines and established the significance of the arginine in the Nef PXXPXR SH3 domain binding motif [20]. The remaining three residues (X’s) were expected to contribute to the specificity of binding for a particular Nef/SH3 domain pair [25-28].

We also searched for functionally significant residues near PQVPLR that could be necessary for Nef/activated PAK2 complex formation, MHCI downregulation, and EVI. Two residues to the N-terminal side P_69_VRPQVPLRP_78_, G67 and F68, were found to be critical for Nef/activated PAK2 complex formation, EVI and MHCI downregulation. Based on our mapping analysis, we have proposed a new multifunctional Nef-host cell protein interface for these activities.

To the C-terminal side of PXXPXR, we also characterized the functional significance of P_78_. In contrast to mutations of PXXPXR, G_67_ and F_68_, the phenotype of the P_78_ mutant was not multiply defective but specific for MHCI downregulation. This result suggests a unique mechanism independent of Nef interacting with an SH3 domain protein. With the current data, we and others have now characterized the dependence of MHCI downregulation on the stretch of Nef amino acids from E_62_ to P_78_ plus the D_123_ residue that is spatially near P_78_[29-33]. These results confirm in detail the recently reported structure for the tripartite complex formed by Nef, AP-1 μ1, and the cytoplasmic tail of MHCI downregulation [34]. Our new results greatly expand the understanding of the polyproline segment, and represent the first advance in integrating Nef structure and function.

## Results

### SH3 domain protein involvement in three Nef activities

The identities of the putative SH3 domain proteins necessary for Nef/activated PAK2 complex formation, MHCI downregulation, and EVI are not known: therefore, the Nef-SH3 domain interactions could only be assessed by functional assays. The three assays were all performed with intact cells which make analysis of the Nef-SH3 domain interaction more difficult to interpret. However, if a direct interaction with an SH3 domain protein is mechanistically required for a given Nef activity, then all three of the point mutations of the prolines and the arginine in PXXPXR should eliminate the activity. If Nef binds multiple SH3 domain proteins, the mutation of the remaining residues PQVPLR, should distinguish between different SH3 domain proteins. Therefore, we have used the requirement that single mutations of both prolines and the arginine are fully defective before concluding that there is a Nef-SH3 domain interaction involved. Conservative mutations of the other three residues of PXXPXR then distinguish between different SH3 proteins that bind Nef [25-27].

### The polyproline helix

We began our investigation of Nef effector interfaces by assaying Nef-Hck SH3 domain binding *in vitro*. The results of these direct binding assays can then be used to evaluate subsequent results of functional assays. The importance of the motif-designating proline residues in PXXPXR have been demonstrated for Nef-Hck SH3 domain binding with the mutations, P72A and P75A, but R_77_ has not been adequately tested [20]. Here, we have used mutations P72G, P75G and R77K. For the other three residues, we mutated Q_73_ to R, V_74_ to I, and L_76_ to V. All of the residues in PQVPLR are very highly conserved (HIV-1 subtype B data base of 1643 sequences, >97% [32]); therefore, even conservative amino acid replacements (as defined by BLOSUM62, [35]) are expected to disrupt of at least one important Nef function. Summarizing, it is expected that the one to one binding of Nef to the Hck SH3 domain will give structurally interpretable information needed to evaluate functional assays.

### Nef-Hck SH3 interaction

**Figure 1:**
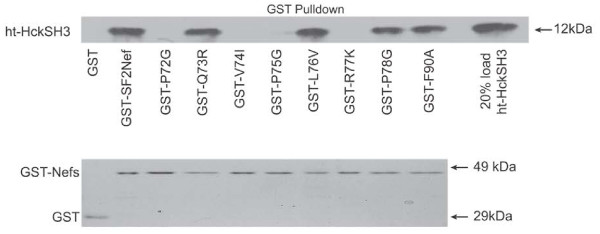
***In vitro*binding to the SH3 domain of ht-HckSH3 by SF2Nef and SF2Nef mutants.** Purified GST and GST-SF2Nef fusion proteins were incubated with the Hck SH3 domain at 3 μM each. The extent of Hck binding to GST or GST-SF2 fusion proteins was assayed as previously described [29]. *Upper Panel*, Bound His-tagged-Hck SH3 domain protein (ht-HckSH3) was determined by Western blot analysis with anti-His tag antibody. After GST-F90A, a lane was skipped and 20% of the amount of ht-HckSH3 was added to the binding reactions and was run in the last lane. *Lower Panel*, Equal amounts of GST and GST fusion proteins were added to the binding assays as shown by SDS/PAGE followed by Coomassie Blue staining.

The ability of the eight singly mutated Nefs to bind to the Hck SH3 domain in direct binding assays is presented in Figure 1. Four of the mutations, P72G, V74I, P75G and R77K each gave no evidence of binding in pull down assays. The results are consistent with X-ray crystal data that P_72_, P_75_, and V_74_ engage in extensive hydrophobic interactions between wild type Nef and the planar hydrophobic surface of the SH3 domain [23,24]. The loss of binding by R77K also reflects the multiple interactions between R_77_ and Hck SH3 residues [23,24]. Conservative mutations of the residues, Q_73_ and L_76_, which do not strongly interact with Hck SH3 domain residues in the crystal structure, did not reduce binding to Hck [23,24]. The disruption of binding by V74I and R77K validates the use of conservative mutations to define Nef interactions with SH3 domain proteins.

Two additional mutations were made to further characterize Nef binding to Hck. In X-ray crystal structures, P_78_ is predicted to bend away from the planar hydrophobic interface of the Hck SH3; and therefore it defines the border of the core SH3 domain binding site [23,24]. As expected, the P78G mutation has minimal impact on Nef-Hck SH3 domain binding. Nef also interacts with the RT loop of the Hck SH3 domain which is outside of the SH3 planar hydrophobic interface [24,36,37]. An isoleucine in the Hck RT loop is buried in a hydrophobic pocket on the surface of Nef formed by L_87_, F_90_, W_113_, I_114_, T_117_ and Q_118_[23,24,38]. Unexpectedly, we found no reduction in binding by the non-conservative F90A mutation (Figure 1). The simplest explanation is that the remaining residues in the pocket are sufficient to stabilize the Nef-Hck SH3 interaction. With these eight mutations, we have a Nef-Hck SH3 domain interaction signature to compare with other Nef activities.

### Nef-SH3 domain interaction required for Nef/activated PAK2 complex formation

Having established a basis for comparing putative Nef SH3 interactions to Nef-Hck SH3 binding, we investigated the role of PQVPLR in Nef/activated PAK2 complex formation. In extracts from cells not expressing Nef, PAK2 autophosphorylation is not detectable by the sensitive *in vitro* kinase activity assay (IVKA) using anti-PAK2 antibody. IVKAs of extracts from cells expressing Nef exhibit both PAK2 autophosphorylation and phosphorylation of the added substrate myelin basic protein ( Additional file 1: Figures S1A and S1B). We have previously demonstrated that in Nef-expressing cells only a small fraction of activated PAK2 is not immunoprecipitated by anti-Nef antibody and that the activation of PAK2 by Nef is sufficient to alter the phosphorylation state of an intracellular protein [39,40]. The components of the Nef/activated PAK2 complex have not been identified leaving open the possibility that the activation of PAK2 by Nef could be a multi-step process with activation separate from complex formation.

To demonstrate a role for an SH3 domain protein in Nef/activated PAK2 complex formation, it is necessary for single mutations of the prolines and the arginine in PXXPXR to abrogate this activity as was observed for Hck binding. It has been previously shown that Nefs with P72A or P75A mutations fail to form the Nef/activated PAK2 complex which suggests SH3 protein involvement [20,41]. However, this suggestion would be called into question if Nef with the R77K mutation gave a functional phenotype. Mutations of the other residues in PQVPLR (Q, V, and L) should give a different mutational profile for Nef/activated PAK2 complex formation compared to Hck (Figure 1).

**Figure 2:**
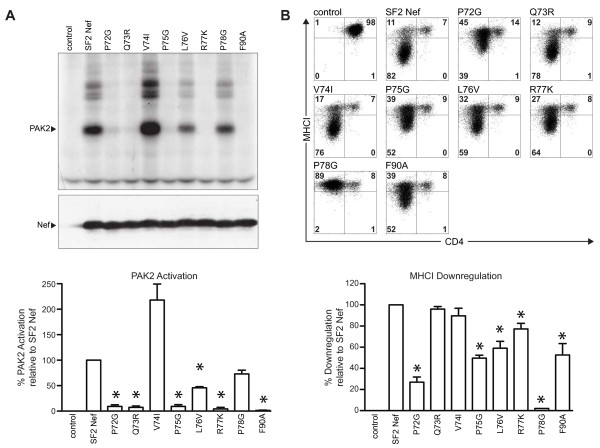
**Mutational analysis of the HIV-1 Nef-SH3 interaction domain: PQVPLR, P**_**78**_**, and F**_**90**_**. (A)***Upper Panelin vitro* PAK2 autophosphorylation assay of anti-Nef immunoprecipitates from transfected 293T cells. Autoradiography shows the autophosphorylated PAK2 band, indicated by the arrow. Higher molecular weight bands that are weakly phosphorylated are proteins associated with the Nef/activated PAK2 complex [43]. *Control* represents transfected 293T cells without Nef expression. *Middle Panel*, Anti-Nef Western blot of whole cell lysates demonstrates equal Nef expression in transfected cells. *Lower Panel*, Phosphoimager quantitation of activated PAK2 relative to SF2Nef set at 100%. Error bars were calculated as the mean ± S.E.M. of three independent experiments. An asterisk indicates a significantly lower value compared to SF2Nef by *t* test (*p* < 0.05). SF2NefV74I and SF2NefP78G are not significantly different from SF2Nef by *t* test (*p* = 0.065 and *p* = 0.065, respectively). **(B)***Upper Panel*, flow cytometric analysis of SF2Nef transduced human CEM T cells. The cell surface expression of CD4 (x-axis) and MHCI (y-axis) was determined as described in Methods. *Control* represents CEM cells transduced without Nef expression. *Lower Panel*, Quantitation of MHCI downregulation relative to SF2Nef is presented. SF2Nef, n = 6, is set at 100%. Error bars were calculated as the mean ± S.E.M. P72G, n = 6; Q73R, n = 4; V74I, n = 4; P75G, n = 6; L76V, n = 4, R77K, n = 4; P78G, n = 3; F90A, n = 3. An asterisk indicates a significantly lower value from SF2Nef by *t* test (*p* < 0.05). Only P78G is not significantly higher than *control* by *t* test.

In Figure 2A, evidence is presented that confirms a role for an SH3 domain protein for Nef/activated PAK2 complex formation. Mutations of the canonical residues of PXXPXR abrogate this Nef activity as was observed for Hck SH3 binding. Strikingly different from the Nef-Hck SH3 domain interaction is the failure of V74I to prevent Nef/activated PAK2 complex formation, and conversely, the elimination of Nef/activated PAK2 complex formation; by Q73R. Thus, conservative mutations of the residues in PQVPLR allow discrimination of the SH3 domain interactions between Nef-Hck and between Nef and the unknown SH3 domain protein required for Nef/activated PAK2 complex formation. L76V, and possibly P78G, have a small negative effect on Nef/activated PAK2 complex formation, but in sharp contrast to Nef-Hck SH3 binding, F90A eliminated it. The drastic effect caused by the F90A mutation suggests an alternate role for F90 distinct from its role as part of the hydrophobic pocket that interacts with the Hck SH3 RT loop. From these observations, we have demonstrated that PQVPLR binds at least two cellular SH3 domain proteins with the canonical residues in PXXPXR. The PXXPXR motif is present in many different cellular SH3 domain binding proteins with different specificities. The non-motif residues (Q73, V74 and L76 in Nef) largely determine which SH3 domain proteins that a particular PXXPXR containing protein will bind [25,26,28]. While Nef exhibits a high degree of specificity for Hck because of its high affinity interaction with Hck, the Nef PXXPXR is also capable of binding to additional SH3 domain proteins at reduced affinity [42].

### Role of PQVPLR, P_78_ and F_90_ in MHCI downregulation

The double mutation of P_72_ and P_75_ to alanines (**A**QV**A**LR) abrogates the ability of Nef to downregulate MHCI [44,45]. It was subsequently reported that the single mutations, P72A and P75A, are only partially defective for MHCI downregulation, but the P78A mutation abolishes this activity [30,33]. Thus, the case can be made that the double mutation of P72A/P75A yields a synthetic phenotype that is the result of a local disruption of Nef structure. In Figure 2B, we evaluated this hypothesis. P_72_ and P_75_ mutated to glycine gave partial defects in MHCI downregulation. Nef with P_78_ mutated to glycine was fully defective for MHCI downregulation (Figure 2B). Significantly, we also demonstrated only a small effect of the R77K mutation on MHCI downregulation (77.3 ± 5.3% of SF2Nef activity), despite the fact that this mutation totally prevents Hck binding and Nef/activated PAK2 complex formation (Figures 1 and 2A). The L76V and F90A mutations also had partial deficits for MHCI downregulation (Figure 2B). Therefore, only the mutation of P_78_ fully abrogates MHCI downregulation in the polyproline region. Our results are suggestive of a relatively non-specific structural role for PQVPLR in MHCI downregulation. CD4 downregulation was also monitored as a positive control of overall Nef activity and was fully functional in all cases. Finally, it should be noted that all the mutations presented in Figure 2 were stably expressed at levels similar to the wild type protein (Figure 2A*Middle Panel*).

Our results do not support the involvement of an SH3 domain protein in MHCI downregulation but point to P_78_ and a nearby residue D_123_. The Nef mutant with D123E is devoid of MHCI downregulation activity [31,32]. The mutational phenotypes of P_78_ and D_123_ are fully consistent with recent proposals of a Nef-induced multi-protein complex that lacks an SH3 domain protein and blocks MHCI transport to the cell membrane [15,18,19,46]. In the Discussion, we review a recent report of the ternary structure of Nef, the cytoplasmic tail of MHCI (ctMHCI) and AP-1 μ1 [34] that is in full accord with the mutational data presented here and previously published [18,19,29,30,32,33].

### Enhancement of virion infectivity (EVI)

**Figure 3:**
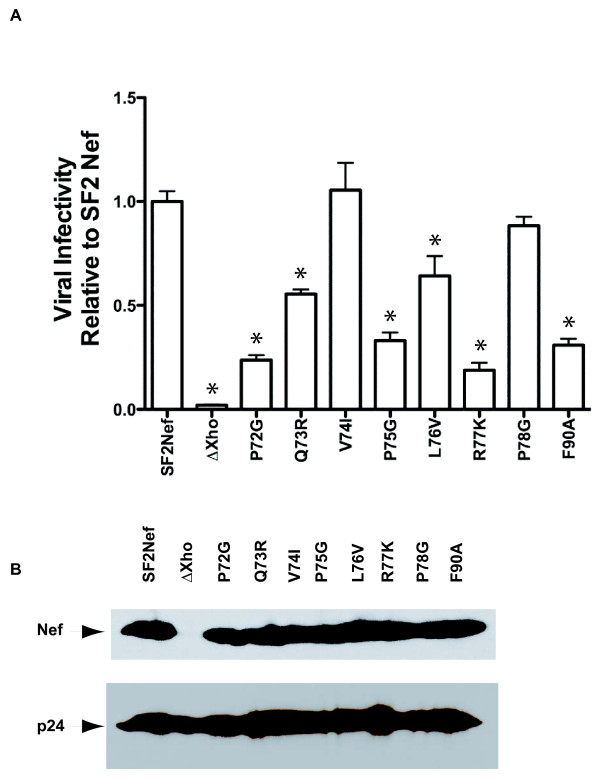
**Mutational analysis of PQVPLR, P**_**78**_**, and F**_**90**_**for Nef-mediated enhancement of virion infectivity. (A)**, HeLa-MAGI cells were infected with HIV-1_SF2_ and HIV-1_SF2_ with mutant *nef*s from Figures 1 and 2. ∆Xho represents HIV-1_SF2_ with its *nef* rendered non-functional by filling in the unique XhoI site with Klenow to make a four base insertion. β-galactosidase positive cells were counted 36 hours post-infection. Viral infectivity (blue cells per ng p24^*gag*^ normalized to HIV-1_SF2_) is presented as the mean ± S.E.M. with n = 6. SF2Nef was set at 100%. An asterisk indicates a significantly lower value compared to SF2Nef by *t* test (*p* < 0.05). Also note that values for all mutated proteins were significantly higher than ∆Xho. **(B)**, Lysates from the 293T producer cells for viruses presented in (A) were analyzed for Nef and p24^gag^ (p24) expression by Western blot. Equal amounts of lysate protein were added to each lane.

Another Nef activity that has been suggested to involve an SH3 binding protein on the basis of the **A**QV**A**LR mutation is EVI [11,47,48]. In Figure 3A, the impact of the mutations from Figures 1 and 2 on this Nef activity is presented. Similar to the results for Nef/activated PAK2 complex formation, P72G, P75G, and R77K, and F90A were all defective for EVI (≤ 33% of SF2Nef activity), but the V74I mutation had no effect (Figure 3A). The L76V mutation of Nef appears to yield partial defects for EVI and Nef/activated PAK2 complex, while the P78G mutant exhibits near wild type activity for EVI and Nef/activated PAK2 complex formation (Figures 2A and 3A). Only Q73R gives a different result in the two assays. Nef mutated to Q73R yields no Nef/activated PAK2 complex but is only 50% defective for EVI (Figures 2A and 3A). We also observed that in contrast to the results for Nef/activated PAK2 complex formation in Figure 2, the impacts of the most debilitating mutations are not complete for EVI (compare SF2NefP72G, P75G, R77K and F90A to SF2Nef∆Xho in Figure 3A). The same residual EVI activity has been reported for the AQVALR double mutation [49]. Finally, the eight Nef mutants are all stable in the context of expression from the SF2 proviral plasmid (Figure 3B). These results suggest that EVI requires an SH3 domain protein that is the same as or similar to the SH3 domain protein involved in Nef/activated PAK2 complex formation.

### The N-terminal side of the polyproline helix

**Figure 4:**
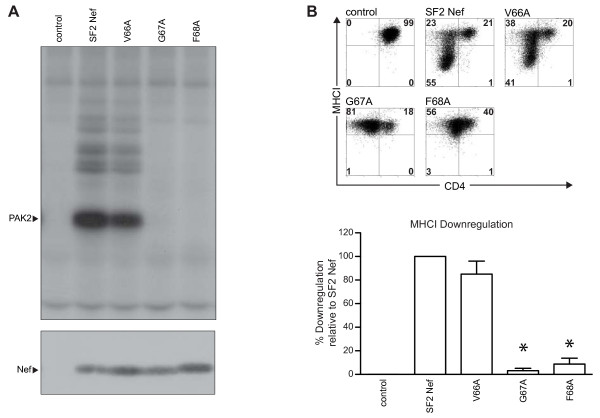
**Functional characterization of residues to the N-terminal side of the Nef polyproline helix: V**_**66**_**, G**_**67**_**, and F**_**68**_**. (A)**, *In vitro* PAK2 autophosphorylation assays were performed on anti-Nef immunoprecipitates from transfected 293T cells. *Upper Panel*, Autoradiography shows the autophosphorylated PAK2 band, indicated by the arrow. *Lower Panel*, Nef expression was determined by anti-Nef Western blot of whole cell lysates. One of two experiments is presented. **(B)**, MHCI downregulation and CD4 downregulation by Nefs from (A). *Upper Panel*, Flow cytometric analysis of Nef-transduced human CEM T cells was performed to assay cell surface expression of CD4 (x-axis) and MHCI (y-axis). *Lower Panel*, Quantitation of MHCI downregulation relative to SF2Nef was set at 100%. Error bars calculated as the mean ± S.E.M. of three independent experiments. An asterisk indicates a significant reduction in activity relative to SF2Nef.

The SH3-binding motif in Nef is flanked by prolines (**P**VRPQVPLR**P**, amino acids 69 and 78). On the C-terminal side, P_78_ is not part of the Nef interface that binds to the Hck SH3 domain. On the N-terminal side, P_69_ has been found to be dispensable for Nef-Hck binding and is, like P_78_, outside of the SH3 binding site of Nef [20,24]. On the other hand, the P69A mutation has been shown to render Nef incapable of Nef/activated PAK2 complex formation [20,41]. Based on these earlier results, we considered the possibility that P_69_ and additional Nef residues just to the N-terminal side of P_69_ constitute an interface that acts in concert with PQVPLR. Specifically, V_66_, G_67_, and F_68_ are three highly conserved residues (99%) that form a loop between the polyproline helix and the tetra-glutamate segment consisting of amino acids 62–65 [32]. In Figure 4, we determined the impact of mutating each of these loop residues to alanine. Similar to P_69_, the adjacent residues, G_67_ and F_68_, are required for Nef/activated PAK2 complex formation while V_66_ is not (Figure 4A *Upper Panel*). G_67_ and F_68_ are also required for MHCI downregulation, but V_66_ is not (Figure 4B). However, there is a clear distinction between MHCI downregulation and Nef/activated PAK2 complex formation in that mutation of P_69_ to alanine does not affect MHCI downregulation [33]. It should be noted that these three Nef mutants were expressed at levels comparable to the wild type protein (Figure 4A *Lower Panel*), and the V66A and G67A mutations had no impact on CD4 downregulation while F68A had only a small effect (72.3 ± 3.2% of SF2Nef activity, See Figure 4B *Upper Panel*). From these results, it is clear that Nef/activated PAK2 complex formation requires the highly conserved residues just to the N-terminal side of the polyproline helix. As these residues are spatially separated from the PQVPLR binding site, it seems likely that G_67_, F_68_ and P_69_, do not interact with an SH3 domain, but instead with a different region of an SH3 domain containing protein or a different protein altogether. Spatially close to the three adjacent amino acids is F_90_. All of these residues (GFP/F) are critical for Nef/activated PAK2 complex formation suggesting that they form an effector domain for this activity (Figures 2A and 4A and [41]). However, MHCI downregulation only requires G_67_ and F_68_ and not P_69_ or F_90_ (Figures 2B and 4B and [33]).

### The impact of the V66A, G67A and F68A mutations on EVI

**Figure 5:**
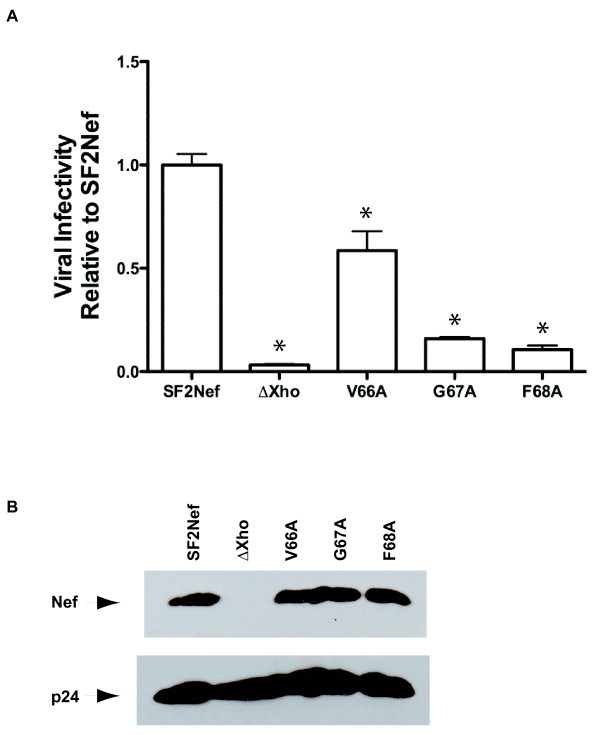
**Impact of V66A, G67A and F68A mutations on Nef-mediated enhancement of virion infectivity. (A)**, HeLa-MAGI cells were infected with HIV-1_SF2_ and HIV-1_SF2_ with mutant *nef*s from Figure 4. ∆Xho represents HIV-1_SF2_ with its *nef* rendered non-functional by filling in the unique XhoI site with Klenow to make a four base insertion. β-galactosidase positive cells were counted 36 hours post-infection. Viral infectivity (blue cells per ng p24^*gag*^ normalized to HIV-1_SF2_) are presented as the mean ± S.E.M. with n = 6. SF2Nef was set at 100%. An asterisk indicates a significantly lower value compared to SF2Nef by *t* test (*p* < 0.05). Also note that values for all mutated proteins were significantly higher than ∆Xho. **(B)**, Lysates from 293T producer cells for the viruses in (A) were analyzed for Nef and p24^gag^ (p24) expression by Western blot. Equal amounts of total lysate protein were added to each lane.

The impact on EVI of mutating the three residues to the N-terminal side of the polyproline helix gave strikingly similar results to the impact on Nef/activated PAK2 complex formation. The V66A mutant retained partial activity while G67A and F68A were maximally defective (Figure 5A). As observed for mutations in PQVPLR, the maximally defective mutations retained residual activity (Figure 3A). The residual activity may reflect a facilitating role for Nef in the case of EVI. Again, in the context of expression from the SF2 provirus the mutant proteins, V66A, G67A and F68A, were stable (Figure 5B).

Qualitatively similar phenotypes for Nef/activated PAK2 complex formation (Figures 2 and 4) and EVI (Figures 3 and 5) suggest interactions by Nef with very similar SH3 proteins, or even the same SH3 protein for these activities. It should be noted that, even if Nef uses the same SH3 protein for Nef/activated PAK2 complex formation and EVI, the overall mechanisms are nonetheless distinct, since EVI is not dependent on Nef/activated PAK2 complex formation [32,50].

### PQVPLR, GFP/F, and P_78_ reside in a region of overlapping, multifunctional effector domains on the surface of Nef

**Figure 6:**
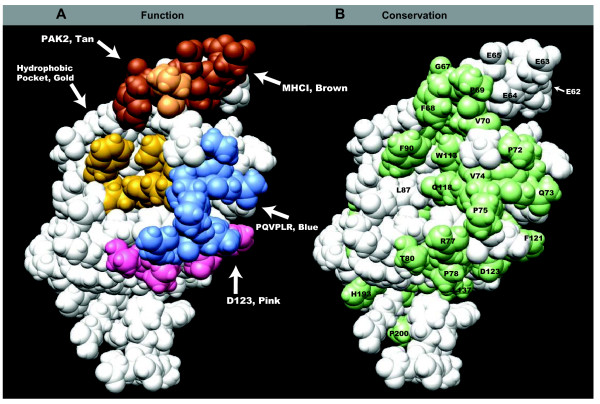
**Organization of effector interfaces on the surface of Nef associated with the polyproline helix. (A)***Left, Function*, The colored residues are grouped into proposed effector interfaces and presented on the three dimensional surface of Nef. The core of the SH3 domain binding interface that includes P_72_, Q_73_, V_74_, P_75_, L_76_, and R_77_ is indicated by “PQVPLR, Blue.” A proposed interaction interface important for MHCI downregulation that includes P_78_ and D_123_ is indicated by “D123, Pink”. Two residues near P_78_ and D_123_ that are 99% conserved, T_80_ and F_121_, are also pink. A second proposed interaction region important for MHCI downregulation is indicated as “MHCI, Brown.” Brown residues include E_62-65_ (tetra-glutamate), G_67_, and F_68_. The hydrophobic pocket that enhances the affinity of Hck binding includes L_87_, F_90_, W_113_, T_117_ and Q_118_ and is indicated with “Hydrophobic Pocket, Gold.” Note: T_117_ is obscured in this view. GFP/F is a proposed interface that overlaps “MHCI, Brown” and “Hydrophobic Pocket, Gold.” PQVPLR and GFP/F act in concert for Nef/activated PAK2 complex formation. GFP/F includes G_67_ and F_68_ (brown), P_69_ (tan), and F_90_ (gold). “PAK2, Tan” indicates P_69_ that is not part of the “MHCI, Brown” interface. White residues remain uncharacterized. **(B)***Right, Conservation*, The same view of Nef as in (A) but the residues that are conserved at 99% in subtype B Nefs are green and labeled. The tetra-glutamate segment (amino acids 62–65) is conserved in the sense of being a negatively charged patch on the surface of Nef, but none of the positions is 99% glutamate. Amino acid 87 that is part of the hydrophobic pocket is 96% leucine, 2% isoleucine and 1% methionine [32]. The modified BH10 Nef (PDB: 2NEF) presented has a truncated N-terminal arm (amino acids 1–55) and has a deletion of the flexible internal loop (amino acids 159–173). These modifications are not visible as amino acids 56, 158, and 174 are on the backside of this view. All images were created using Pymol (www.pymol.org).

The spatial relationships between the residues in the polyproline helix and the surrounding region of Nef are shown in Figure 6A *Function*. PQVPLR is in blue, GFP/F is in gold (F_90_), brown (G_67_ and F_68_) and tan (P_69_), P_78_ and D_123_ plus nearby highly conserved residues are in pink; and the hydrophobic pocket containing F_90_ is in gold. In HIV-1 subtype B Nefs, only about 45% of residues are conserved at the 99% level. In Figure 6B *Conservation*, residues that are labeled and colored green are 99% conserved in HIV-1 subtype B Nefs (L_76_ is 97% conserved [32]). The proximity of these amino acids on the surface of Nef and their extreme conservation are consistent with roles in functionally significant protein-protein interactions [51].

Each of the colors represents potential multifunctional interaction domains for Nef binding to cellular proteins. Brown residues are important for MHCI downregulation. These include the tetra-glutamate segment (residues E_62_-E_65_), G_67_ and F_68_. The quadruple mutation of the tetra-glutamate segment to four alanines has been reported by many groups to be defective for MHCI downregulation [15,19,29,44,45]. The proximity of G_67_ and F_68_ to the tetra-glutamate segment suggests concerted action by these residues in MHCI downregulation.

G_67_ and F_68_ are also required for Nef/activated PAK2 complex formation and EVI. P_69_ is included in this patch as tan because it contributes to Nef/activated PAK2 complex formation, but not MHCI downregulation. Gold residues have a role in Hck binding [23,24,38,52]. Here, we report for the first time the importance of F_90_ for Nef/activated PAK2 complex formation and EVI. To reflect the overlapping functionalities of its residues we represented GFP/F in three different colors (Figure 6A).

Blue residues are Nef’s multifunctional PXXPXR motif, and pink residues surround P_78_ and D_123_ (Figure 6A). D_123_ is multifunctional and required for MHCI downregulation, EVI and CD4 downregulation [31,32,53]. The conservative mutation D123E is fully defective for these three Nef activities and enhances Nef/activated PAK2 complex formation several fold [31,32]. This latter effect may be related to the fact that mutating the nearby F121 to arginine prevents Nef/activated PAK2 complex formation [20]. D_123_, P_78_, T_80_, and F_121_ residues are part of a predicted protein-protein interaction interface [54]. The mechanism of action of D_123_ for different functions has remained obscure, although the recent report of the ternary complex of Nef, AP-1 μ1 and ctMHCI explains the roles of P_78_ and D_123_ in MHCI downregulation [34].

## Discussion

### Identification of critical amino acids in Nef protein-protein interaction interfaces

We have carefully mapped by mutational analysis residues that could possibly act in concert with PQVPLR in Nef-SH3 domain protein interactions. Mutations were found that resulted in partial defects, and mutations were found that eliminated one or more Nef activities. Mutations of the latter phenotype were observed for G_67_, F_68_, P_72_, V_74_, P_75_, R_77_, P_78_, and F_90_. The mutational properties of these residues fit the expected properties of critical residues in protein-protein interfaces. Typically, critical residues in protein-protein interfaces exhibit packing densities equivalent to that found in the interiors of proteins which account for the large disruption of binding by a conservative replacement or by creating a hole in the interface with an alanine substitution (glycine excepted). Further, these critical amino acids are typically found in the central core of protein-protein interfaces [51]. For a given interface only a few of the residues account for most of the binding affinity, and identifying these amino acids localizes the interface [51,55,56].

### The multifunctional PXXPXR motif

For Nef binding to the Hck SH3 domain, four critical residues (P_72_, V_74_, P_75_, and R_77_) have been defined by mutation (Table 1). These four residues engage in multiple interactions with Hck SH3 amino acids. Numerous residues that weakly contribute to Nef-Hck SH3 binding have also been noted [23,24,27,52]. For Nef/activated PAK2 complex formation, it is the mutation of P_72_, Q_73_, P_75_ or R_77_ (but not V_74_) that gives a total loss of function phenotype with a similar phenotypic profile observed for EVI. Therefore, we conclude that the Nef PXXPXR motif is multifunctional and can bind more than one SH3 domain protein.

**Table 1:** Summary of phenotypes for mutations in the Nef polyproline region

**Residue**	**Hck SH3**	**PAK2**	**MHCI**	**EVI**	**CD4**
**EEEE/AAAA**	#	+^1^	−^1^	+^1^	++^1^
**V66A**	#	+	++	+	++
**G67A**	#	−	−	−	++
**F68A**	#	−	−	−	+
**P69A**	++^3^	−^3,5^	++^6^	−^5^	++^6^
**P72G**	−	−	+	−	++
**Q73R**	++	−	++	+	++
**V74I**	−	++	++	++	++
**P75G**	−	−	+	+	++
**L76V**	++	+	+	+	++
**R77K**	−	−	++	−	++
**P78G**	++	+	−	++	++
**F90A**	++	−	+	+	++
**D123E**	#	++^2,4^	−^2,4^	−^4^	−^2,4^

Results are presented from this report unless otherwise indicated. ^1^Baugh *et al*. [29]; ^2^Kwak *et al*. [31]; ^3^Manninen *et al*. [20]; ^4^O’Neil *et al*. [32]; ^5^Wiskerchen *et al*. [41]; ^6^Yamada *et al*. [33]. Hck SH3, *in vitro* binding of Nef to Hck SH3 domain; PAK2, Nef/activated PAK2 complex formation; MHCI, MHCI downregulation; EVI, enhancement of virion infectivity; CD4, CD4 downregulation. #, indicates that the residue is outside of the known Nef-Hck SH3 interface [23,24]; “++” activity of mutated protein is >75% of wild type protein; “+” activity is between 25% and 75% of wild type; “-“ activity is less that 25% of wild type. EEEE/AAAA is the quadruple mutation of the tetra-glutamate segment (amino acids 62–65) of Nef mutated to four alanines.

### There are overlapping effector surfaces at the N-terminal end of the polyproline helix

We have defined a new interface, GFP/F, which is necessary for Nef/activated PAK2 complex formation and full activity of EVI (Table 1). In Figure 6A *Function*, GFP/F is presented in three colors with G_67_ and F_68_ (brown) residing between F_90_ (gold) and P_69_ (tan). We propose that GFP/F is part of a protein–protein interaction interface for Nef/activated PAK2 complex formation based on our mutational analysis and the 99% conservation of these residues [32]. To test this model we made the conservative mutation, F68Y, and we found this mutant Nef to be fully defective for Nef/activated PAK2 complex formation ( Additional file 2: Figure S2A). The most straightforward explanation for this result is that F_68_ is a critical residue for Nef/activated PAK2 complex formation engaging in hydrophobic interactions which the extra hydroxyl group of tyrosine disrupts.

A recently proposed interface overlapping GFP/F is the secretion modulation region (SMR, spanning V_66_GFPV_70_ but not including F_90_). Bond and co-workers have shown that secretion of Nef in exosomes and the binding of Nef to mortalin depends on this motif. *In silico* modeling suggests the SMR motif forms a protein binding pocket [57,58]. Therefore, there appear to be at least four Nef functions modulated by this region of the Nef surface including MHCI downregulation, Nef/activated PAK2 complex formation, mortalin binding and EVI but not Hck binding [59].

### A multifunctional surface at the C-terminus of the polyproline helix

P_78_ and D_123_ Nef mutants are defective for MHCI downregulation (Table 1). D_123_ represents a multifunctional residue as the conservative mutation, D123E, is highly defective for MHCI downregulation, CD4 downregulation and EVI. A computer analysis of the surface of Nef (PPI-Pred) included P_78_ and D_123_ along with T_80_ and F_121_ as part of a putative protein-protein interaction site [54]. All of these residues (pink in Figure 6A) are highly conserved [32]. Further D_123_ and F_121_ are part of a proposed dimerization interface [53,60,61]. This region of the Nef surface appears to resemble the region at the N-terminus of the polyproline helix in its multifunctionality.

### MHCI downregulation

MHCI is a common target for anti-host defenses of human viruses. HIV-1 Nef counters MHCI directed cytoxicity in a distinct mechanism from those of adenovirus, human CMV and Kaposi’s sarcoma-associated herpesvirus and even simian immunodeficiency virus Nef [62,63]. Key to HIV-1 Nef’s mechanism is residue P_78_. P_78_ appears to be specifically involved in MHCI downregulation as mutating P_78_ to alanine has little or no effect on other Nef activities (Figures 2,3 and [20,30,33,41,58]. The recent description of a ternary structure of ctMHCI, AP-1 μ1 and Nef (http://retroconference.org/2012b/Abstracts/45450.htm) strongly implies that P_78_ is critical for Nef binding and rerouting MHCI away from the cell surface [34].

An extended stretch of Nef amino acids from E_62_ to P_78_ binds to both AP-1 μ1 and ctMHCI in a manner entirely different from the binding of Nef to SH3 domains [23,24,34]. In the model of Jia *et al*., the tetra-glutamate segment (E_62_E_63_E_64_E_65_) electrostatically interacts with a patch of four lysines in AP-1 μ1. This ionic interaction is consistent with our previous report that several combinations of the tetra-glutamate segment mutated to two glutamates and two alanines all remain functional for MHCI downregulation [29]. In the new structure, G_67_ and F_68_ are part of a bend in the Nef main chain that allows the polyproline helix to be positioned in the complex near the YXXL binding site of AP-1 μ1. The polyproline segment of Nef serves as a clamp over the low affinity combination of ctMHCI bound to AP-1 μ1. ctMHCI binds weakly to the YXXL site of AP-1 μ1 because the cryptic motif in ctMHCI is the defective YXXA. Down the length of the polyproline segment the interactions between Nef residues and the complex are not extensive. Therefore, the structure rationalizes the partial defects observed with single mutations of PXXPXR residues while the double mutation of AXXAXR is fully defective [15,19,29,44,45]. P_78_ is the exceptional residue in that it is buried in the ternary complex. Spatially near P_78_ is D_123_, which is also critical for MHCI downregulation [31,32,53]. D_123_ forms a salt bridge with R_393_ of AP-1 μ1 as part of a network of electrostatic interactions between all three proteins. Therefore, the model predicts the mutational defects seen in MHCI downregulation with P78G and D123E.

The ternary complex model originally proposed by Guatelli and co-workers and Collins and co-workers [15,18,19,46], has been contested as others have argued for involvement of SH3 domain host cell proteins, specifically Hck, Lyn and Src or alternatively a decisive role for Nef dimerization that is dependent on residue D_123_[21,44,53,61]. SH3 domain binding is not part of the new structure, and the role of D_123_ in the new structure excludes it from participating in dimerization.

The structure of Jia *et al* is a major advance but important questions remain unanswered [34]. Residue M_20_ is analogous to P_78_ in its significance and specificity for MHCI downregulation ( Additional file2: Figure S2B and [19,64,65]). Here we have found G_67_ and F_68_ to also be structurally required for MHCI downregulation. These Nef residues may be involved in binding additional factor(s) to the Nef effector complex for MHCI downregulation [46].

### Analysis of the *in vivo* significance of Nef activities

Detailed characterization of Nef protein-protein interaction interfaces makes feasible targeted studies of the impact of Nef activities on HIV-1 replication and cytotoxic impact *in vivo*. BLT humanized mice represent an advanced model for reconstitution of the human immune system [66-68]. We have recently published the complex phenotype of *nef*(-)HIV-1 in this system of reduced viral replication and highly cytotoxic effects on T cells and thymocytes [69]. Significance for a given Nef activity can be established by mutations specific for that function that result in either reduced HIV-1 replication/cytotoxicity or rapid reversion of the mutation.

It should also be noted that a second recent report investigated the three amino acid loop, V_66_, G_67_, and F_68_[70]. Mutations of these amino acids resulted in defects for MHCI downregulation, Nef/activated PAK2 complex formation, EVI but not CD4 downregulation. In addition, the Nef activities of inhibition of actin remodeling following T cell receptor stimulation, hyper-phosphorylation of cofilin and CXCR4 downregulation were dependent on the loop residues.

## Conclusions

We have undertaken detailed mutational analysis of polyproline helix and proximal surface residues that are critical for multiple Nef activities. Four of the Nef activities that have been suggested to be dependent on host cell SH3 domain proteins have been investigated. We have demonstrated that three Nef activities, Nef/activated PAK2 complex formation, Hck binding and EVI are dependent on a Nef-SH3 domain protein interaction. When subjected to detailed analysis a fourth activity, MHCI downregulation, does not exhibit the properties expected of an activity dependent on an SH3 domain protein. Particularly telling in this regard is the small impact of the R77K mutation on MHCI downregulation. Furthermore, the three Nef activities that definitely require SH3 domain proteins do not depend on the same host cell protein. Hck and at least one additional SH3 domain protein interact with Nef.

The functionally dense region of the Nef surface investigated here serves as a model for the protein’s structural complexity. The stretch of amino acids from E_62_ to P_69_ plus the spatially close F_90_ contains at least four combinations of residues that effect separate Nef activities-Nef/activated PAK2 complex formation, EVI downregulation, MHCI downregulation, and mortalin binding. From P_72_ to R_77_, PQVPLR acts as a PXXPXR motif to bind at least two different SH3 domain proteins and contributes to MHCI downregulation. Near the C-terminal end of the polyproline helix D_123_ is involved in at least three different Nef activities [31,32]. For MHCI downregulation, D_123_ acts in conjunction with P_78_ to pin the ctMHCI to AP-1 μ1. The rest of the polyproline segment plays a supporting role in ternary complex by positioning the tetra-glutamate segment to interact with a lysine patch on AP-1 μ1.

Maintenance of the structure of this critical region of Nef enforces strict conservation of the component residues and implies that the associated activities confer fitness to the virus. Extending the type of analysis presented here to the rest of the Nef surface will define which Nef activities are sufficiently important to structurally impose overlapping protein-protein interaction interfaces and the attending extreme conservation of amino acid residues. Detailed functional analysis will allow activity-specific mutations in Nef to be evaluated in BLT humanized mice. Highly conserved protein-protein interaction interfaces for significant Nef activities would then be preferred targets for developing drug-like inhibitors of Nef function [71].

## Methods

### Cell lines and culture conditions

Human CEM T cells were cultured in RPMI 1640 medium supplemented with 10% fetal bovine serum (Hyclone), 50 IU of penicillin per ml, 50 μg streptomycin per ml, 2 mM L-glutamine and 1 mM sodium pyruvate. HEK 293T cells were cultured in DMEM with 10% fetal bovine serum, 50 IU of penicillin per ml, 50 μg/ml of streptomycin and 2 mM L-glutamine. HeLa-MAGI cells were cultured in the presence of 1.0 mg/ml puromycin. Cell lines were maintained at 37°C in a humidified incubator with 5% CO_2_ (CEM) or 10% CO_2_ (293T and HeLa-MAGI).

### Plasmid DNA constructs

The pcDNA3.1-SF2Nef plasmid DNA expression vector was utilized for transient transfection of 293T cells. Bacteria expression vectors were pGEX2TSF2Nef which expresses full length Nef as a GST fusion protein and pET28aHckSH3 which expresses amino acids 61–117 of Homo sapiens Hck (gi:56203176) in frame with polyhistidine. The pLXSN, pLNefSNSF2 (wild type or mutated), and pEQPAM vectors were transfected into HEK 293T cells to generate amphotropic vectors for subsequent transduction of CEM T cells. The p9B18 HIV-1_SF2_ proviral clone was transfected into HEK 293T producer cells to generate infectious virus for HeLA-MAGI infectivity assays. Point mutations were made by site-directed mutagenesis in SF2Nef as previously described [72]. All mutations were verified by DNA sequencing and are numbered in accordance with the standard Nef length of 206 amino acids.

### Transfection of HEK 293T cells

One day prior to transfection, HEK 293T cells were plated at 800,000 cells per well in six-well plates such that the cells would be 90% confluent upon transfection. The pcDNA3.1, pcDNA3.1-SF2Nef, p9B18-SF2, pLXSN and pEQPAM plasmid DNA vectors were transfected into the cells using Lipofectamine 2000 (Invitrogen) according to the manufacturer’s instructions. Four hours post-transfection, the culture medium was replaced with fresh DMEM with 10% fetal bovine serum and antibiotics. 36–48 hours post-transfection, cell lysates and/or virus supernatants were harvested for subsequent experiments.

### Transduction of human CEM T cells

pEQPAM (2 μg) and pLNefSNSF2 (2 μg) were co-transfected into HEK 293T cells to generate amphotropic virus as described above. Harvested viral supernatants were filtered through 0.45 μm filters. 24-well plates were coated with 400 μg of Retronectin (Takara) followed by 500 μl of 2% bovine serum albumin in phosphate buffered saline. Wells were then rinsed and virus was absorbed upon incubation with 500 μl of virus supernatant twice at one-hour intervals. CEM T cells (300,000 per well) were incubated overnight. The next day, a third addition of 500 μl of viral supernatant was added on top of the cells. Twenty four hours later, cells were collected, washed and re-suspended in complete RPMI containing 1.5 mg/ml G418 for selection. Cells were expanded into T-75 cm^2^ flasks, at which point the cells were harvested for subsequent Western blot analysis, *in vitro* kinase assay, and flow cytometric analysis.

### Western blot analysis and antibodies

Cells were washed in phosphate-buffered saline and lysed in 1 ml of Buffer A (50 mM Tris, pH = 8.0, 100 mM NaCl, 25 mM NaF, 25 mM benzamidine, 20 mM β-glycerophosphate, 2 mM Na_3_VO_2_, 3 mM EDTA, 10% glycerol, and 0.5% IGEPAL-630) containing one Roche protease inhibitor tablet per 10 ml. Lysates were centrifuged at 13,000 X g for 30 minutes and the supernatant fraction was prepared for SDS-PAGE gel electrophoresis. Separated proteins were transferred to nitrocellulose. The transferred blots were blocked with 10% milk and subsequently incubated with primary antibodies for one-hour at room-temperature, washed, and probed with HRP-conjugated secondary antibodies. Western blots were visualized by chemiluminescence upon incubation with luminol and *p*-coumaric acid. Nef protein expression was determined using the sheep polyclonal anti-SF2Nef serum, as previously described [32,72].

### *In vitro* kinase assay

Endogenous Nef/activated PAK2 complex formation assays were performed as previously described [32,72]. Nef-expressing 293T cells were lysed in Buffer A containing one Roche protease inhibitor tablet per 10 ml. Lysate containing 600 μg of protein was completely immunoprecipitated with 10 μl sheep anti-SF2 Nef serum and protein A agarose beads (Sigma) [39]. In one experiment ( Additional file 1: Figure S1A), 10 μl of anti-PAK2 antibody (Cell Signaling) was used. Protein A beads were washed twice in lysis buffer, once in 1M MgCl_2_, and twice in kinase buffer (10 mM Tris, pH = 7.5, 5 mM MgCl_2_ and 1% Triton X-100). The beads were then resuspended in 100 μl of kinase buffer. γ-^32^P]-ATP was incubated with the immunoprecipitates for 10 minutes at room temperature. The kinase reaction was terminated upon addition of EDTA. Proteins were eluted with SDS Laemmli buffer and resolved by 9% SDS-PAGE. Phosphorylated proteins were quantitated by phosphoimager analysis (Packard Cyclone). In Additional file 1: Figure S1B, the combined assay of PAK2 atuophosphorylation and myelin basic protein phosphorylation was performed in identical fashion to the autophosphorylation assay except that 15 μg of myelin basic protein were added to the 0.1 ml autophosphorylation reaction.

### Flow cytometry analysis

For analysis of cell surface CD4 and MHCI levels, transduced CEM cells (500,000) were first incubated with mouse monoclonal anti-haplotype A1, A11, and A26 MHC class I antibody (One Lambda) for 20 minutes on ice and then washed. Cells were then incubated with fluorescein isothiocyanate (FITC)-labeled rabbit anti-mouse IgG for 20 minutes on ice and then washed. Cells were then incubated with 2 μg of mouse IgG for 20 minutes on ice and then washed. Cells were then incubated with phycoerythrin (PE)-conjugated IgG1 monoclonal antibody to human CD4 (Exalpha) for 20 minutes on ice and then washed. Stained cells were analyzed on a BD FACSCalibur instrument equipped with CellquestPro software. CEM cells transduced with LXSN served as the positive control. For negative controls mouse isotype antibody replaced anti-MHC class I, and PE-conjugated mouse IgG1 (Exalpha) replaced PE-conjugated anti-CD4.

### HeLa-MAGI infectivity assays

As described above, HEK 293T cells were transfected with the proviral clone for SF2 (p9B18) to generate infectious virus. Virus was harvested 48 hours post-transfection, filtered through a 0.45-μm filter, and stored frozen in aliquots at −70°C. The virus produced was normalized by p24^gag^ ELISA. HeLa-MAGI cells (80,000) were plated in 12-well plates and infected 24 hours later in triplicate with 5 ng of p24^*gag*^ of virus in 400 μl of cell culture medium containing 20 μg/ml of DEAE-dextran (Sigma) at 37°C. After a two-hour incubation, fresh culture medium was added, and the cells were incubated for an additional 36 hours. The cells were then fixed and stained with X-gal. Blue cells were counted as indicators of infected cells. Expression of Nef and p24^gag^ in lysates of 293T producer cells was analyzed by Western blot analysis as described above.

### *In vitro* binding assay

BL-21 pLysS bacteria were transformed with pGEX4T, pGEX2TSF2Nef, pGEX2TSF2NefP72G, pGEX2TSF2NefQ73R, pGEX2TSF2NefV74I, pGEX2TSF2NefP75G, pGEX2TSF2NefL76V, pGEX2TSF2NefR77K, pGEX2TSF2NefP78G, pGEX2TSF2NefF90A, and pET28HckSH3 (Hck amino acids 61-117). The fusion protein derived from pGEX2TSF2Nef has been previously shown to bind the SH3 domain of Hck in pulldown assays [29]. A 10 ml starter culture in Luria-Bertani medium with 34 μg/ml chloramphenicol and either 50 μg/ml of ampicillin (pGEX construct) or kanamycin (pET28 construct) was initiated from a single colony, grown at 37°C 225 RPM, and used to inoculate 250 ml of Luria-Bertani medium with antibiotics as described previously [29]. The 250 ml culture was grown at 37°C, 225 RPM until optical density at 600 nm reached 0.6. Isopropyl-ß-D-thiogalactopyranoside was added to the culture to a final concentration of 3 mM and allowed to induce for 3 hours shaking at 37°C and 225 RPM. Bacteria were pelleted and stored frozen at -20°C.

Bacteria pellets containing glutathione S-transferase (GST) fusion proteins or histidine (His) tagged protein were resuspended in 15ml of PBS containing one tablet of Roche protease inhibitor per 10 ml. The resuspended bacteria were sonicated on ice using a microtip for 1 minute at power level 3.5; 10s on and 10s off. Bacterial lysates were centrifuged at 10,500 RPM, 4°C, 20 minutes. Supernatants of GST fusion proteins were rotated with 0.5 ml packed glutathione Sepharose beads (GE Healthcare) for 30 min at room temperature on a rotator. His-tagged protein was incubated with 0.5 ml packed HisPur Cobalt Resin (Pierce) for 30 minutes at 4°C on a rotator. At 4°C, the Sepharose beads were washed with 10ml cold PBS three times, and HisPur cobalt resin was washed with 6ml HisPur wash buffer (50 mM sodium phosphate, 300 mM NaCl, and 10 mM imidazole) three times. The bound GST fusion protein was eluted with 20 mM reduced glutathione pH 8.0, and the bound His-tagged protein was eluted with HisPur elution buffer (50 mM sodium phosphate, 300 mM NaCl, 150 mM imidazole). Concentration of all proteins was determined by Bradford; purity was determined by SDS-PAGE electrophoresis; and each protein was exhaustively dialyzed in binding buffer (50 mM Tris pH7.5, 1% IGEPAL CA-630, 2 mM MgCl_2_, 200 mM NaCl and 5 mM ß-mercaptoethanol). Binding assays with proteins at 3 μM were performed as described previously [29] and analyzed by Western blotting with polyclonal anti-polyhistidine (Abcam).

## Competing interests

The authors declare that they have no competing interests.

## Authors’ contributions

LSK, LLB, SJD, RLW and ML performed experiments and made Figures. JVG and JLF designed experiments and wrote the manuscript. All authors read and approved the final manuscript.

## Supplementary Material

Additional file 1 **Figure S1. Activated PAK2 cannot be detected in 293T cells without Nef expression. (A)**, *Upper Panel*, The autophosphorylation activity of SF2Nef was determined as in Figure 2 except that PAK2 was directly immunoprecipitated by anti-PAK2 antibody. The autophosphorylated band of PAK2 is indicated by an arrow. Vector control not expressing SF2Nef is indicated by “pcDNA.” Note the total absence of activity in the control cells without Nef. *Lower Panel*, Anti-Nef Western demonstrating expression of SF2Nef. **(B)**, The *in vitro* kinase assay was performed on anti-Nef immunoprecipitates with the sole modification that 15 μg of myelin basic protein was added to the assay. *Upper Panel*, PAK2 is the autophosphorylated PAK2 protein, and MBP is the phosphorylated myelin basic protein. *Middle Panel*, Coomassie blue stain of MBP in the reaction. *Lower Panel*, Anti-Nef Western blot demonstrating expression of SF2Nef. Click here for file

Additional file 2 **Figure S2. Nef/ activated PAK2 complex formation is defective for SF2NefF68Y but wild type for SF2NefM20A. (A),***Upper Panel*, The activities of SF2Nef and the four indicated SF2Nef mutants were determined as in Figure 2. The autophosphorylated band of PAK2 is indicated by an arrow. Vector control not expressing SF2Nef is indicated by “pcDNA.” *Lower Panel*, Anti-Nef Western demonstrating equal expression of SF2Nef and the mutated proteins. **(B)**, Same as in (A) for vector control, SF2Nef and SF2NefM20A. Click here for file
