# Supplementary material for: Overlapping effector interfaces define the multiple functions of the HIV-1 Nef polyproline helix
Source: Retrovirology. 2012 May 31;9:47. doi: 10.1186/1742-4690-9-47 (PMC3464899; doi:10.1186/1742-4690-9-47)
Supplement: Additional file 1 — Figure S1. Activated PAK2 cannot be detected in 293T cells without Nef expression. (A), Upper Panel, The autophosphorylation activity of SF2Nef was determined as in Figure 2 except that PAK2 was directly immunoprecipitated by anti-PAK2 antibody. The autophosphorylated band of PAK2 is indicated by an arrow. Vector control not expressing SF2Nef is indicated by “pcDNA.” Note the total absence of activity in the control cells without Nef. Lower Panel, Anti-Nef Western demonstrating expression of SF2Nef. (B), The in vitro kinase assay was performed on anti-Nef immunoprecipitates with the sole modification that 15 μg of myelin basic protein was added to the assay. Upper Panel, PAK2 is the autophosphorylated PAK2 protein, and MBP is the phosphorylated myelin basic protein. Middle Panel, Coomassie blue stain of MBP in the reaction. Lower Panel, Anti-Nef Western blot demonstrating expression of SF2Nef. [file 1742-4690-9-47-S1.pdf]

Figure S1

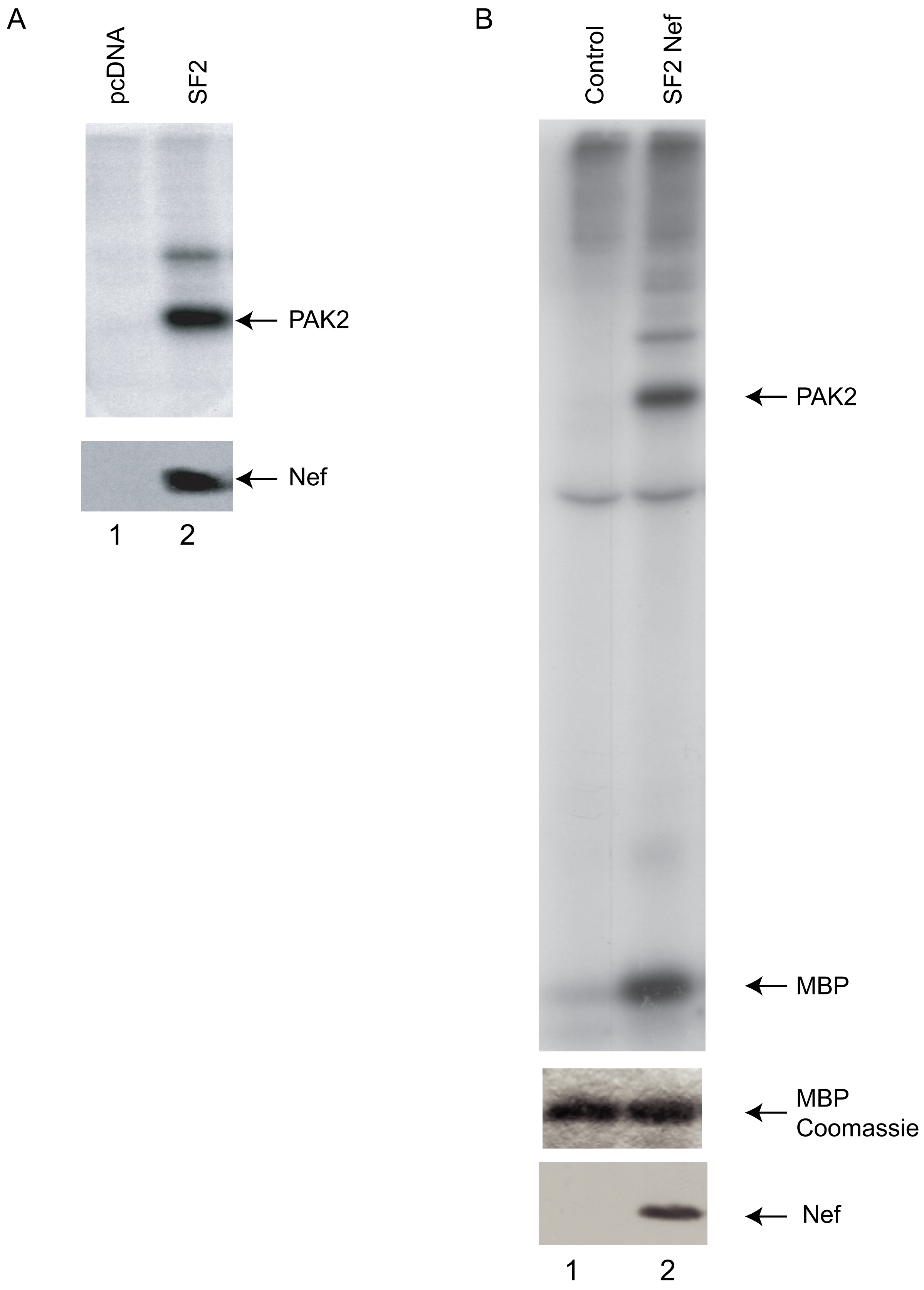

Figure S1. SF2 induces the activation of PAK2 autophosphorylation and transkinase phosphorylation.
