# Supplementary figures and images for: Overlapping effector interfaces define the multiple functions of the HIV-1 Nef polyproline helix
Source: Retrovirology. 2012 May 31;9:47. doi: 10.1186/1742-4690-9-47 (PMC3464899; doi:10.1186/1742-4690-9-47)

**Figure S2**

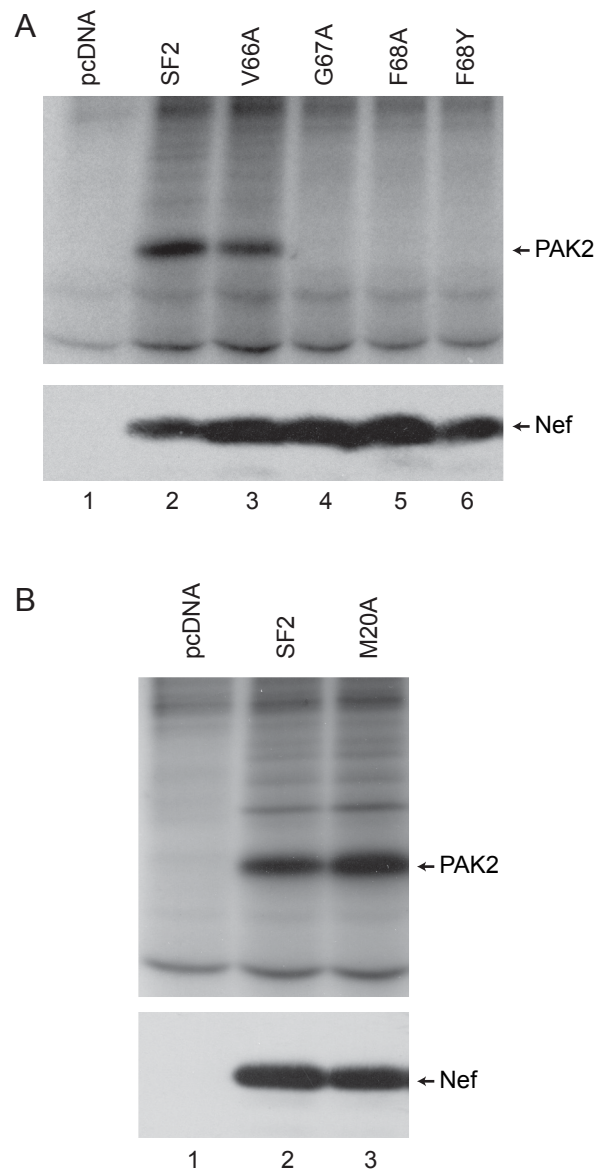

**Figure S2. PAK2 activation is defective for SF2NefF68Y but wild type for SF2NefM20A.**

Supplement: Additional file 2 — Figure S2. Nef/ activated PAK2 complex formation is defective for SF2NefF68Y but wild type for SF2NefM20A. (A),Upper Panel, The activities of SF2Nef and the four indicated SF2Nef mutants were determined as in Figure 2. The autophosphorylated band of PAK2 is indicated by an arrow. Vector control not expressing SF2Nef is indicated by “pcDNA.” Lower Panel, Anti-Nef Western demonstrating equal expression of SF2Nef and the mutated proteins. (B), Same as in (A) for vector control, SF2Nef and SF2NefM20A. [file 1742-4690-9-47-S2.pdf]
